# Conflicting invasive electrophysiological study results in a suspected fasciculoventricular pathway: a case report

**DOI:** 10.1093/ehjcr/ytag103

**Published:** 2026-02-06

**Authors:** Alexander Welcker, Djemail Ismaili, Andreas Rillig, Feifan Ouyang

**Affiliations:** Department for Invasive Electrophysiology, Department for Cardiology, University Medical Center Hamburg-Eppendorf, Martinistraße 52, Hamburg 20246, Germany; Department for Invasive Electrophysiology, Department for Cardiology, University Medical Center Hamburg-Eppendorf, Martinistraße 52, Hamburg 20246, Germany; German Center for Cardiovascular Research (DZHK), Partner Site Hamburg/Lübeck/Kiel, Hamburg, Germany, Martinistraße 52, Hamburg 20246, Germany; Department for Invasive Electrophysiology, Department for Cardiology, University Medical Center Hamburg-Eppendorf, Martinistraße 52, Hamburg 20246, Germany; German Center for Cardiovascular Research (DZHK), Partner Site Hamburg/Lübeck/Kiel, Hamburg, Germany, Martinistraße 52, Hamburg 20246, Germany; Department for Invasive Electrophysiology, Department for Cardiology, University Medical Center Hamburg-Eppendorf, Martinistraße 52, Hamburg 20246, Germany; German Center for Cardiovascular Research (DZHK), Partner Site Hamburg/Lübeck/Kiel, Hamburg, Germany, Martinistraße 52, Hamburg 20246, Germany

**Keywords:** Case report, Pre-excitation, Accessory pathway, Fasciculoventricular pathway, Nodoventricular pathway, Mahaim fibres

## Abstract

**Background:**

Fasciculoventricular pathways (FVPs) are variants of pre-excitation syndrome with uncertain prevalence in the human heart. This case report presents findings from an invasive electrophysiological study (EPS) that mostly fulfil the established diagnostic criteria for FVP, but also exhibit atypical features, highlighting diagnostic ambiguity in clinical practice.

**Case summary:**

A 23-year-old patient presented with a history of brief, weekly episodes of palpitations. The resting surface ECG demonstrated a pre-excitation pattern suggestive of either a parahisian or fasciculoventricular pathway. The EPS findings were largely consistent with a FVP. During junctional beats, we observed a loss of pre-excitation and normalization of HV-interval. No tachycardia was inducible, and there was no evidence of retrograde conduction via an accessory pathway.

**Discussion:**

Three potential anatomical locations of the upper FVP take-off side were considered: (i) a single FVP originating from the lower part of the His-bundle or (ii) a single FVP originating close to the upper, junctional part of the His-bundle or even from the distal part of the AV-node and (iii) the presence of an additional second accessory pathway. Based on the above-mentioned evidence, the second anatomical location of the upper FVP-insertion was most likely, differing from previous findings of FVP in the literature. A conservative, observational management strategy was adopted. Current diagnostic criteria for FVP are based on retrospective studies and case reports, lacking prospective validation. This case underscores the need for further research to refine the diagnostic approach to FVP and improve risk stratification in such patients.

Learning points

- Fasciculoventricular pathways (FVPs) are a variant of pre-excitation, typically arising from the lower His-bundle and inserting in the crest of the muscular ventricular septum.
- The typical electrophysiological study characteristic is among others a prolongation of the AH-interval with a constant shortened HV-interval (<35 ms) during incremental atrial pacing.
- This report discusses an atypical take-off side of a FVP close to the junctional part of the His bundle and the clinical reasoning for an observational strategy because of the benign characteristics of FVPs.

## Introduction

Fasciculoventricular pathways (FVPs) are accessory fibres connecting the atrioventricular (AV) conduction axis to the crest of the muscular ventricular septum, first described by Mahaim.^1^ Their true prevalence remains uncertain. Electrophysiological studies (EPS) in adult cohorts report rates of 1.2%–1.8%,^2,3^ whereas histological investigations identified FVPs more frequently in human cardiac anatomy.^4^ An ECG-based survey in children found FVPs to be the most common form of ventricular pre-excitation, accounting for 76.7% of observed cases.^5^ This discrepancy may be attributed to factors such as the subtle amount of pre-excitation of FVPs on surface-ECGs, absence of arrhythmias in isolated FVPs, and variability in their anatomic and electrophysiological characteristics.^2,4^

As FVPs are located distal to the AV-node (AVN), they neither support AV re-entrant tachycardia nor conduct rapid atrial arrhythmias to the ventricles. Consequently, they are not associated with an increased risk of sudden cardiac death.^6^ However, it is clinically significant to distinguish isolated FVPs from cases where they coexist with other pathways, as this can alter risk stratification and therapeutic decision-making.^2^ This report describes a case of suspected FVP in which one diagnostic criterion was not fully met, and we outline our clinical reasoning in navigating this diagnostic ambiguity.

## Summary figure

**Table:** Current diagnostic criteria of FVP (selective literature research)

**Surface-ECG characteristics indicating FVP**	**Invasive EPS characteristics of FVP**
Low delta wave amplitude (≤2 mm) in the most pre-excited frontal limb lead QRS, 40 ms after onset^6^	HV-interval during sinus rhythm <35 ms^7^
PR-interval around 100–120 ms^3,6,8^	Prolongation of the AH-interval with constant HV-interval during incremental atrial pacing^2,7,8^
QRS-duration ≤120 ms (100–120 ms)^3,6,8^	Constant degree of pre-excitation and constant shortened HV-interval independently to the origin of excitation (including parahisian pacing), unless the pacing or extrasystole occurs below take-off of the FVP^2,4,8^
Constant degree of pre-excitation under adenosine-induced AV-block^2,4^	Concentric and decremental retrograde atrial activation during incremental ventricular pacing^3,8^
Angle between QRS and delta wave axis around 25° to differentiate from a parahisian pathway (around 3°)^7^	No inducible re-entry tachycardia^3,8^

## Case presentation

**Figure 1:**
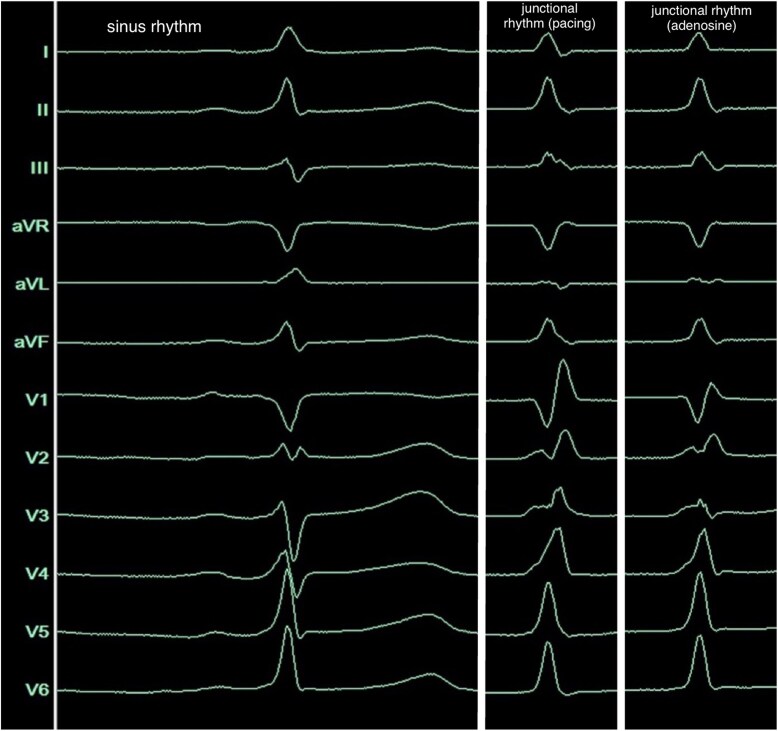
Twelve-lead surface-ECG (100 mm/s) during sinus rhythm (left), during junctional rhythm, reached by incremental atrial pacing (middle) and under the administration of adenosine (right).

A 23-year-old female patient was referred to our university rhythm outpatient clinic due to previously noticed pre-excitation in her surface-ECG. She reported episodes of palpitations lasting several minutes, occurring approximately twice weekly over the past 2 years. These episodes were self-limiting, with no clear correlation to physical exertion or rest. No ECG documentation of the palpitations had been obtained to date. The patient’s medical history was unremarkable. Initial non-invasive evaluations—including exercise ECG (except fixed QRS-duration of 105 ms, irrespectively of the cycle length of sinus rhythm)—revealed no abnormalities. Given the persistence of symptoms and evidence of ventricular pre-excitation on the surface-ECG (*Figure 1*), an invasive EPS was scheduled.

**Figure 2:**
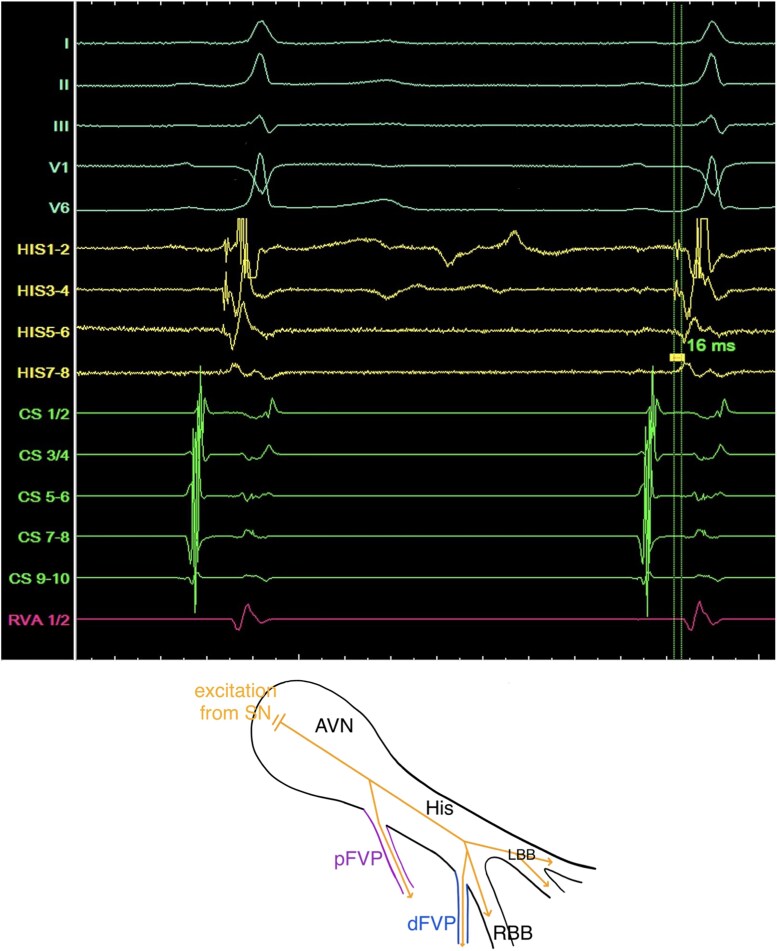
Top: Intracardiac signals and five leads of the surface-ECG in sinus rhythm with a shortened HV-interval of 16 ms. Bottom: Schematic drawing of the AVN/HP-system with a simultaneously portrayal of a proximal FVP (pFVP, purple) or distal FVP (dFVP, blue) during sinus rhythm.

**Figure 3:**
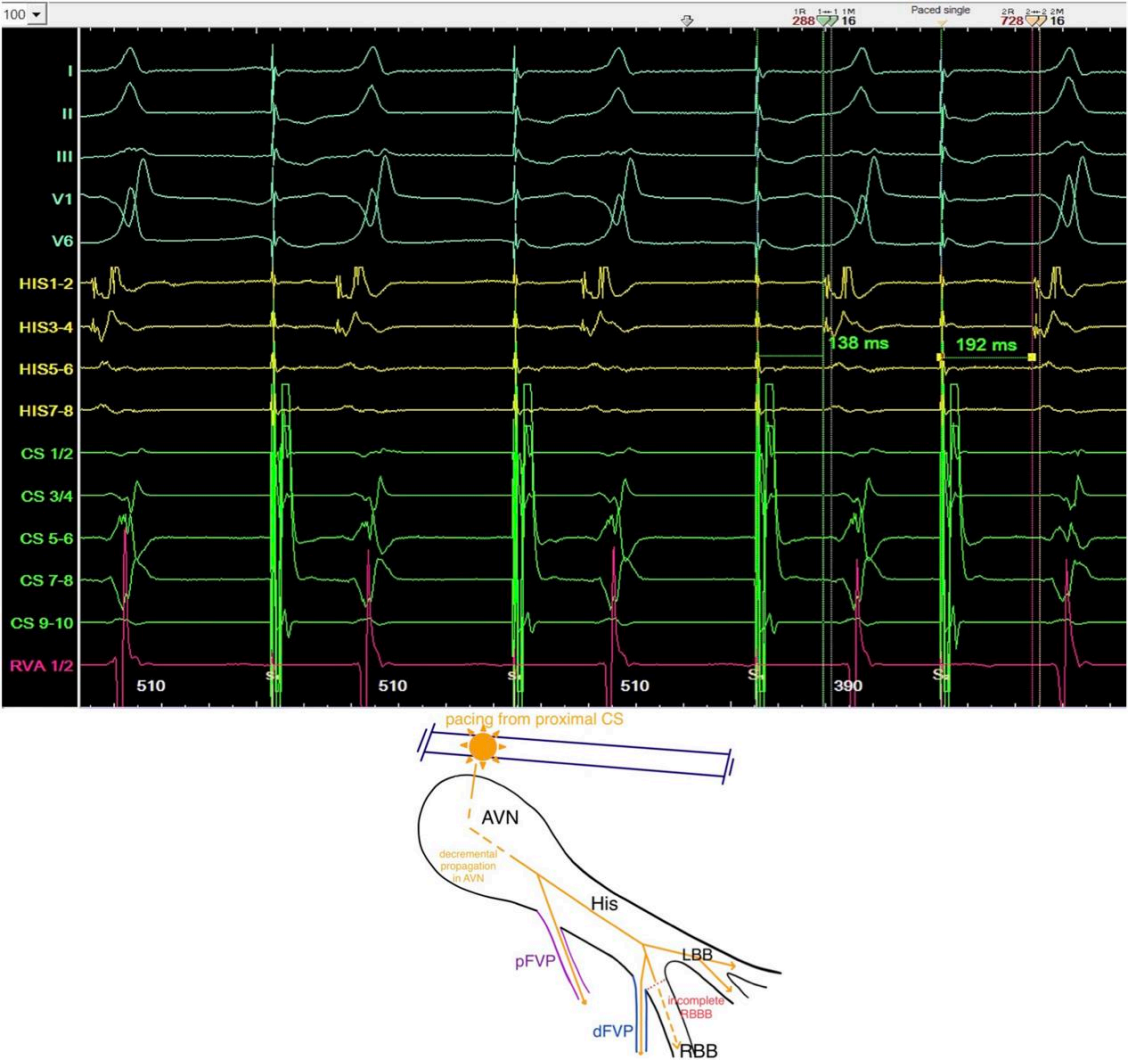
Top: Antegrade programmed stimulation from the proximal CS-catheter with prolongation of the AH-interval but constant HV-interval. Bottom: Schematic drawing of the AVN/HP-system with a simultaneously portrayal of a proximal FVP (pFVP, purple) and distal FVP (dFVP, blue) during CS-pacing.

**Figure 4:**
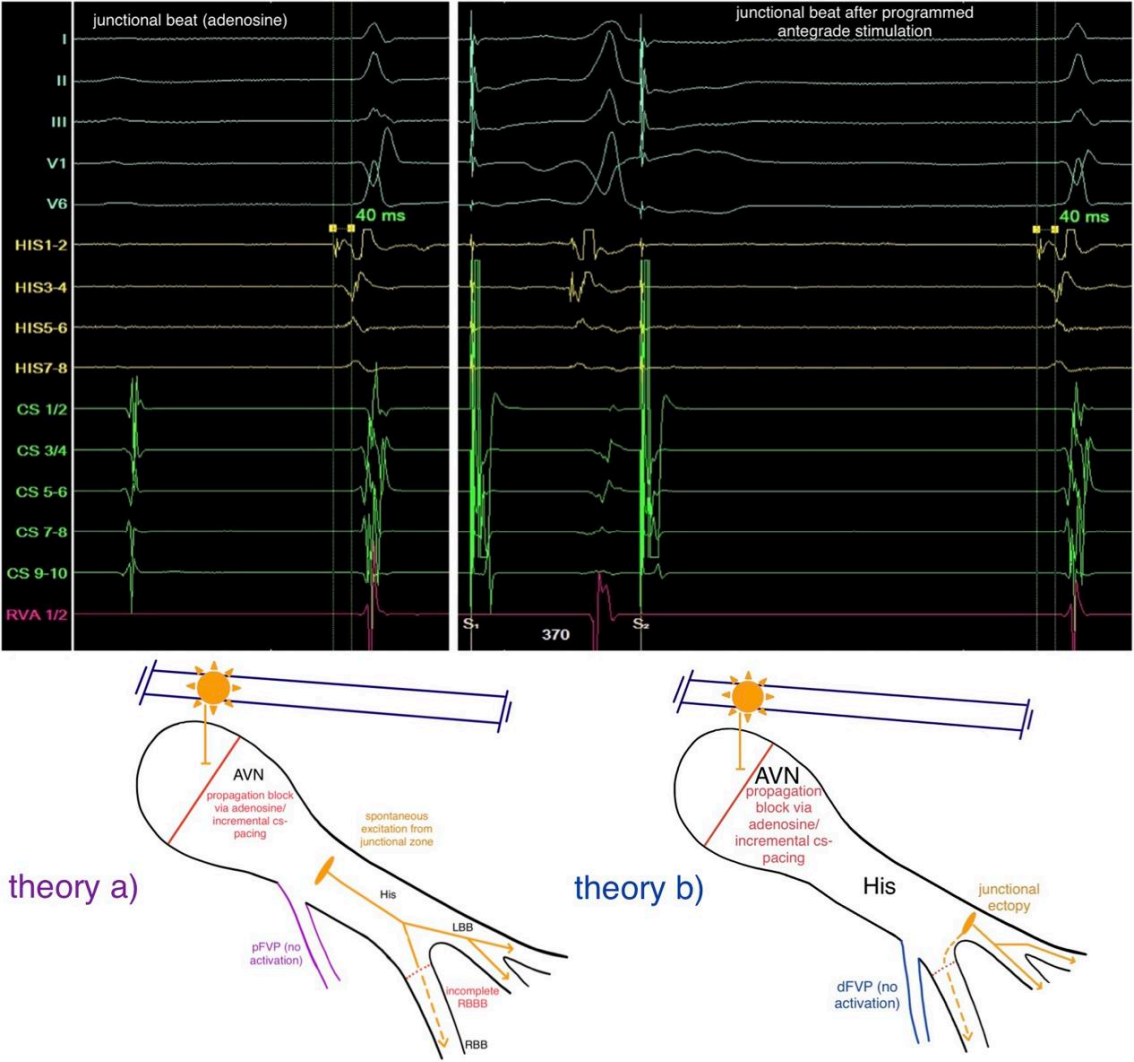
Top: Junctional beats with normalization of the HV-interval achieved with the administration of adenosine (left side) and during programmed antegrade stimulation with a S1S1/S1S2-interval of 510/370 ms (right side). Bottom: Two schematic drawings of the AVN/HP-system how the loss of pre-excitation and normalization of the HV-interval during junctional beats can be explained with a proximal FVP (theory a, purple, pFVP) and a distal FVP (theory b, blue, dFVP) by junctional ectopy.

**Figure 5:**
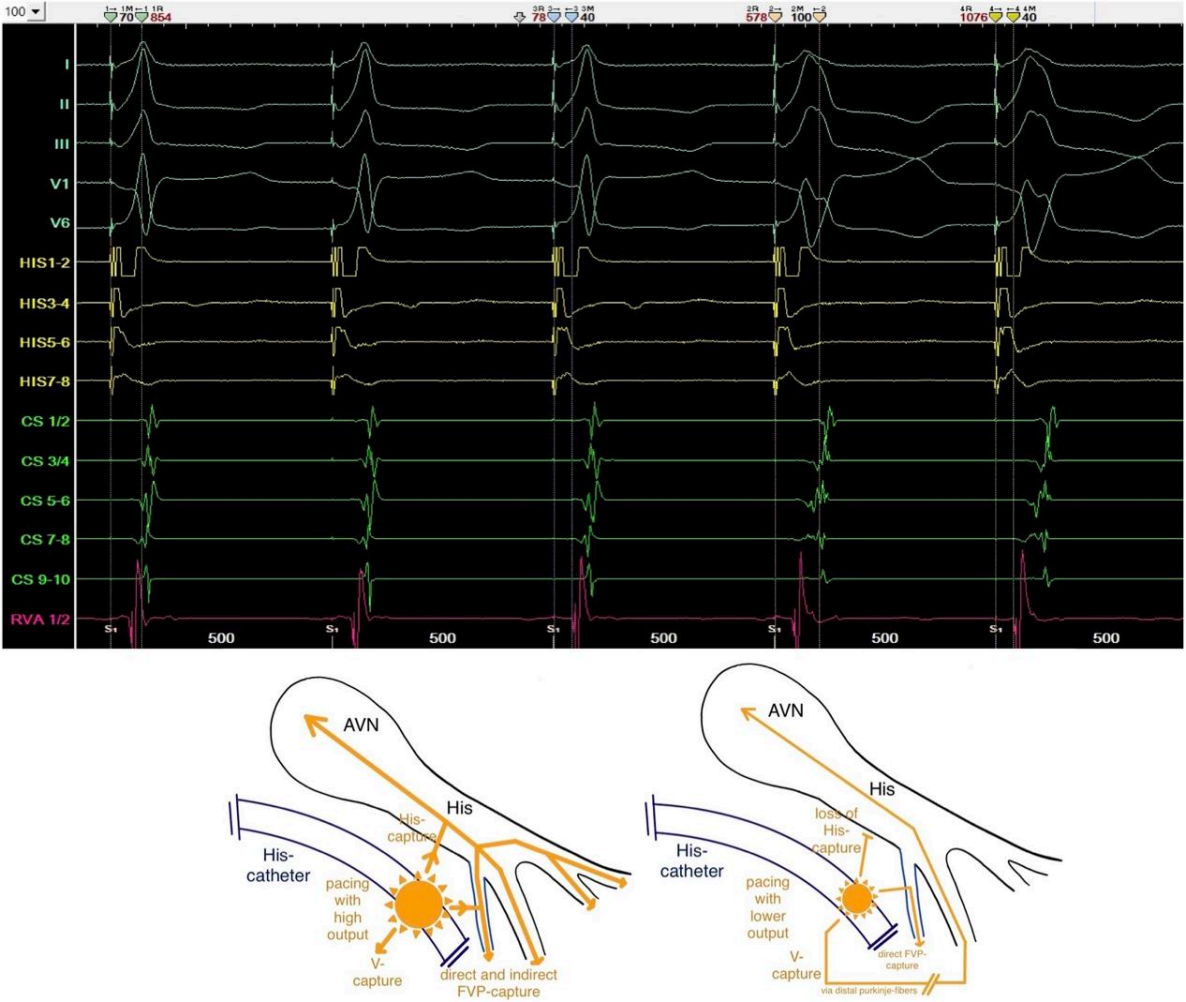
Top: Parahisian pacing showing identical QRS-morphology during the thirst three beats as observed in sinus rhythm. Green and orange marker sets labelling the increasing SA-intervals from narrow to broader QRS-complexes; blue and yellow marker sets labelling the constant S-RVA-intervals. Bottom: Two schematic drawings of the AVN/HP-system representing how the parahisian pacing manoeuvre is supporting a distal FVP take-off side, assuming His-capture (left side) during the narrow and pure V-capture (right side) during the broad QRS-complexes.

The EPS was performed under analgosedation (propofol, fentanyl), supplemented by local anaesthesia at the right femoral access site. Three diagnostic catheters were inserted via the right femoral vein and positioned in the coronary sinus (CS), right ventricular apex (RVA), and His-bundle/right-bundle-branch (RBB) region. Baseline measurements demonstrated a shortened HV-interval (16 ms) with a normal AH-interval (*Figure 2*). Retrograde conduction exhibited physiologically concentric and decremental atrial activation. During programmed antegrade stimulation from the proximal CS-catheter, the AH-interval prolonged while the HV-interval remained constant (16 ms) (*Figure 3*). With a S1S1/S1S2-interval of 510/370 ms, we observed a normalization of the HV-interval (40 ms) and loss of pre-excitation with an incomplete RBB-block (RBBB) QRS-morphology, indicating the antegrade effective refractory period of the pathway (*Figure 4*). Under the administration of 16 mg adenosine, achieving junctional rhythm, the same QRS-morphology, normalized HV-interval and loss of pre-excitation as observed during programmed antegrade stimulation were registered (*Figures 1* and *4*). Parahisian pacing with His-capture produced a QRS-morphology identical to that observed during sinus rhythm and during assumed ventricular (V)-capture a prolongation of stimulus to atrial activation compared to His-capture (*Figure 5*). No tachyarrhythmias were inducible during the EPS, including with and without the administration of isoprenaline as well as atropine.

## Discussion

Based on the morphology of the surface-ECG, a parahisian accessory pathway was initially suspected (*Figure 1*). This interpretation was supported by the application of established algorithms designed to localize antegrade-conducting pathways according to their pre-excitation patterns.^9–11^ Intracardiac signals obtained during sinus rhythm demonstrated a shortened HV-interval and earliest ventricular activation near the RBB regarding the signals from the RV-, HIS-, and CS-catheters, prompting consideration of a FVP (*Figure 2*). This hypothesis was further supported during incremental stimulation from the proximal CS-catheter, which revealed prolongation of the AH-interval while the HV-interval remained consistently shortened (*Figure 3*). Assuming that junctional beats typically originate from the upper portion of the His-bundle, specifically the transitional region between the AVN and the His-bundle, and considering the histological findings reported by Gormel and Yasar^3^ which describe FVPs as typically arising from the lower His-bundle, our observations during junctional rhythm, namely, the loss of pre-excitation and a normalized HV-interval, were somewhat contradictory (*Figures 1* and *4*).

A junctional ectopy originating from the left fascicular system, distal to the typical take-off site of a FVP, may explain this conflict. The incomplete RBBB-morphology of the junctional beats supports this interpretation; however, the preceding His-potential and normalized HV-interval contradict it (*Figures 1* and *4*).

Parahisian pacing with assumed simultaneously His- and V-capture (narrow QRS during the first three beats in *Figure 5*), producing the same morphology of pre-excitation as during sinus rhythm (*Figures 1* and *5*), supported the typical take-off origin of the FVP and the ventricular insertion close to the midseptal area near the right-bundle-branch.^2,4^ But the consistent stimulus-to-earliest-RVA (S-RVA) intervals during both narrow and broad QRS-complexes (*Figure 5*) limit the interpretive value of the manoeuvre in this case: it remains unclear whether the broader QRS-complexes represent pure V-capture with direct activation of the FVP, supporting a typical take-off site or unselective His-capture (His+V) with indirect activation of the FVP via the His-Purkinje-system (HPS) and selective His-capture (without local V-capture) during the narrow QRS-complexes, not clearly supporting a distal take-off origin but explaining constant S-RVA-intervals.^12,13^

Due to these findings, we discussed a proximal (atypical) take-off origin of the FVP, close to the junctional part or even in the lower part of the compact AVN, making it a nodoventricular accessory pathway.^7^ A detailed analysis of the His-channel recordings in *Figure 2* demonstrates a proximal (HIS7–8) to distal (HIS1–2) ventricular activation sequence occurring simultaneously with the sharp His-potential. Furthermore, during incremental atrial pacing (*Figure 3*), the developed incomplete RBBB could potentially affect the HV-interval if the FVP originated distally (fascicular). However, the HV-interval remained constant with a prolongation of the AH-interval (*Figure 3*), strongly supporting an atypical take-off site. The tracings during parahisian pacing with His-capture would therefore only be compatible with capture of a broader region due to high stimulation output, unselective His-Capture instead of pure V-capture during the beats with broader QRS-complexes or retrograde activation of the FVP.^12,13^

The possibility of a second accessory pathway was considered. However, the concentric, decremental retrograde conduction during incremental RV-pacing and the prolongation of stimulus-to-atrial-activation intervals with loss of His-capture during parahisian pacing rendered retrograde conduction through a second accessory pathway unlikely.

Considering the overall findings, absence of ECG documentation of symptomatic episodes, failure to induce tachycardia during the EPS, and the proximity to the specific AV-conduction system, the decision was made to conclude the procedure without mapping and ablation of the ventricular insertion of the FVP. The patient was recommended a wearable to document symptomatic episodes and was discharged the following day.

Based on the findings of Macías *et al*. and Suzuki *et al*., FVPs may be more prevalent than previously thought.^4,5^ Consequently, it might appear or overseen more frequently in EPS, highlighting the need for validated criteria to differentiate FVPs from accessory pathways capable of re-entry or 1:1 atrial tachycardia conduction—conditions that pose greater risk than FVP alone. Current criteria are derived from case series and retrospective studies, lacking prospective validation. This report underscores the difficulty of clinical decision-making with the existing criteria, emphasizing the need for further investigation in this field.

## Lead author biography


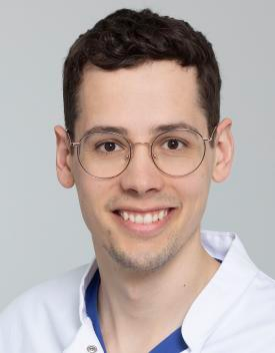


The lead author is a young resident physician in speciality training for cardiology with interest in invasive electrophysiology. He practices at the University Heart and Vascular Center Hamburg.

## Data Availability

All figures presented in the case report are available upon request to the corresponding author.

## Notes

**Consent:** The authors confirm that written informed consent for submission and publication of this case report including images and associated text has been obtained from the patient, in line with COPE guidance.
